# Prediction of Phytochemicals for Their Potential to Inhibit New Delhi Metallo β-Lactamase (NDM-1)

**DOI:** 10.3390/ph16101404

**Published:** 2023-10-03

**Authors:** Zainab Bibi, Irfa Asghar, Naeem Mahmood Ashraf, Iftikhar Zeb, Umer Rashid, Arslan Hamid, Maria Kanwal Ali, Ashraf Atef Hatamleh, Munirah Abdullah Al-Dosary, Raza Ahmad, Muhammad Ali

**Affiliations:** 1 Department of Biotechnology, Abbottabad Campus, COMSATS University Islamabad, Abbottabad 22060, Pakistanchishti@cuiatd.edu.pk (R.A.); 2 School of Biochemistry and Biotechnology, University of Punjab, Lahore P.O. Box 54590, Pakistan; naeem.sbb@pu.edu.pk; 3 Department of Chemistry, Abbottabad Campus, COMSATS University Islamabad, Abbottabad 22060, Pakistan; umerrashid@cuiatd.edu.pk; 4 LIMES Institute, University of Bonn, D-53113 Bonn, Germany; ahamid@uni-bonn.de; 5 Institute of Nuclear Medicine, Oncology and Radiotherapy (INOR), Abbottabad 22060, Pakistan; maria.kanwal.ali@outlook.com; 6 Department of Botany and Microbiology, College of Science, King Saud University, P.O. Box 2455, Riyadh 11451, Saudi Arabia; ahatamleh@ksu.edu.sa (A.A.H.); almonerah@ksu.edu.sa (M.A.A.-D.)

**Keywords:** New Delhi metallo β-lactamase-1, phytochemicals, polyphenols, molecular dynamics simulations, metallo β-lactamase inhibitors

## Abstract

The effectiveness of all antibiotics in the β-lactam group to cure bacterial infections has been impaired by the introduction of the New Delhi Metallo-β-lactamase (NDM-1) enzyme. Attempts have been made to discover a potent chemical as an inhibitor to this enzyme in order to restore the efficacy of antibiotics. However, it has been a challenging task to develop broad-spectrum inhibitors of metallo-β-lactamases. Lack of sequence homology across metallo-β-lactamases (MBLs), the rapidly evolving active site of the enzyme, and structural similarities between human enzymes and metallo-β-lactamases, are the primary causes for the difficulty in the development of these inhibitors. Therefore, it is imperative to concentrate on the discovery of an effective NDM-1 inhibitor. This study used various in silico approaches, including molecular docking and molecular dynamics simulations, to investigate the potential of phytochemicals to inhibit the NDM-1 enzyme. For this purpose, a library of about 59,000 phytochemicals was created from the literature and other databases, including FoodB, IMPPAT, and Phenol-Explorer. A physiochemical and pharmacokinetics analysis was performed to determine possible toxicity and mutagenicity of the ligands. Following the virtual screening, phytochemicals were assessed for their binding with NDM-1using docking scores, RMSD values, and other critical parameters. The docking score was determined by selecting the best conformation of the protein–ligand complex. Three phytochemicals, i.e., butein (polyphenol), monodemethylcurcumin (polyphenol), and rosmarinic acid (polyphenol) were identified as result of pharmacokinetics and molecular docking studies. Furthermore, molecular dynamics simulations were performed to determine structural stabilities of the protein–ligand complexes. Monodemethylcurcumin, butein, and rosmarinic acid were identified as potential inhibitors of NDM-1 based on their low RMSD, RMSF, hydrogen bond count, average Coulomb–Schrödinger interaction energy, and Lennard–Jones–Schrödinger interaction energy. The present investigation suggested that these phytochemicals might be promising candidates for future NDM-1 medication development to respond to antibiotic resistance.

## 1. Introduction

β-lactam antibiotics are the most widely used antibiotics; however, they are vulnerable to inactivation by a growing number of β-lactamases [1]. The essential β-lactam ring of these antibiotics is hydrolyzed by the β-lactamase enzyme, making it difficult for the antibiotics to bind with their drug targets in the pathogen [2]. Multidrug resistance has become a significant concern to world health as a result of the advent and spread of superbugs [3,4]. Recently, there has been a lot of concern about multi-drug resistance in Gram-negative bacteria [5]. Many infectious diseases, including tuberculosis and cholera that had been controlled in developed countries are recurring due to an alarming increase in antibiotic resistance [6]. Among various β-lactamases, NDM-1 (New Delhi Metallo-β-lactamase) has prime importance due to its broad substrate specificity and wide spread [7]. Serine-β-lactamases (SBLs) and metallo-β-lactamases are the two primary families of β-lactamases that have been identified based on the mechanism of enzyme catalysis [8]. Additionally, based on similarities in their structures and sequences, these enzymes have been further divided into the A, B, C, and D classes. Class B enzymes are zinc-dependent hydrolases, whereas class A, C, and D enzymes are serine β-lactamases because they utilize a catalytic serine residue as the reactive nucleophile [9,10]. The enzyme NDM-1 has been classified as a class B metallo-enzyme based on catalytic activity and sequence similarity.

The emergence of the NDM-1 enzyme from class B beta-lactamases has made β-lactam antibiotics less effective in treating various infections. The NDM-1 enzyme was first discovered in *Klebsiella pneumoniae,* isolated from the clinical sample of a Swedish patient who was previously treated in New Delhi, India [11]. After NDM-1 was identified, its variants started to appear all over the world, forcing the World Health Organization to issue a global alert [7]. The NDM-1 gene has been identified in a variety of bacterial pathogens, including *Acinetobacter baumannii*, *Escherichia coli*, *Enterobacter cloacae*, *Klebsiella pneumoniae*, and *Pseudomonas aeruginosa* [12,13,14,15]. Structurally, NDM-1 is a member of the MBL family’s subclass B1. The active site of NDM-1 is surrounded by a flexible loop and contains two zinc ions that are connected by a hydroxide ion [16]. The amino acids His120, His122, and His189 and a hydroxide ion bind to the first zinc ion in the active site of NDM-1, while the amino acids Asp124, Cys208, and His250 bind to the second zinc ion [16,17]. The first zinc ion aligns with the carbonyl group of the substrate for nucleophilic attack, whereas the second zinc ion interacts with the amide nitrogen and carboxyl group, both of which are β-lactam antibiotics properties. The nucleophilic attack on β-lactam rings is caused by the hydroxide, which finally leads to substrate hydrolysis [7].

Due to the flexible active site and high catalytic efficiency of the enzyme, the efficacy of most of the β-lactam antibiotics, such as penicillin, cephalosporins, and carbapenems, has been impeded [18,19,20]. Variants of NDM-1 have been emerging around the globe, and gene encoding of this enzyme is frequently carried by plasmids [21]. Acknowledging the reported studies, NDM-1 is a principle contributor of the clinical threat; hence, designing a potent inhibitor would be helpful in combating this global threat [22]. 

The hydrolytic nature of zinc ligands, catalytic processes, and rapid changes in the active site layout have been the major constraints in the development of NDM-1 inhibitors. Only a few inhibitors have been discovered, including Captopril, Aspergillomarasmine, and Thiorphan [23,24,25,26]. However, no clinically available inhibitor exists to treat NDM-1-resistant bacterial strains, owing to the physiochemical nature of these inhibitors and safety concerns [19]. Captopril, which is a renowned blood pressure medication, chelates to the zinc ion, which is required for the normal functioning of the various human enzymes, such as the angiotensin-converting enzyme [27]. This drug has been shown to be an effective MBL inhibitor as a result of its capacity to chelate zinc ions via the free thiol group. D-captopril and L-captopril inhibit metallo-β-lactamases by using their free thiol group to displace the water molecule that bridges the two zinc atoms in the enzyme’s active site [28]. However, focusing on metal chelators is not a good practice, because mutations in allosteric site residue sequences in some MBLs can significantly reduce the enzymes’ reliance on zinc ions [28]. 

Plant metabolites have been used to treat a variety of infectious diseases. Attempts are being made to identify the inhibitors from natural products and traditional medicines. Many databases have been created with the goal of gathering phytochemicals from various food and medicinal plants [29,30]. These databases provide an ample source of phytochemicals for the purpose of virtual screening. Use of secondary metabolites of plants with antimicrobial properties would be effective in therapeutic treatments [31]. It is worth mentioning that β-lactamase inhibitors should be used in combination with β-lactam antibiotics to treat infections, as they can restore the antibacterial activity of the antibiotics. In silico strategies, like molecular docking, molecular dynamics simulations, and pharmacokinetics, can be particularly helpful for screening a large number of phytochemicals. These methods can forecast how a protein and its ligand will interact at a molecular level [32,33]. These techniques can anticipate the binding affinity, molecular interactions, and physiochemical characteristics of the hit compounds, saving time and money [32]. Previously, a number of in silico studies have been conducted using molecular docking and molecular dynamics simulations for the discovery of potent inhibitors of β-lactamases, particularly NDM-1, from various natural resources [34,35,36,37]. These studies have identified many phytochemicals with different chemical structures and properties that can inhibit NDM-1 [38,39], but none of them have yet been approved for use in humans. Screening these large libraries of phytochemicals to discover inhibitors of NDM-1 could lead to the identification of new and more potent drug candidates. The current study sought to identify potent metabolites that could act as natural NDM-1 inhibitors.

## 2. Results

### 2.1. Cross-Validation

The results of cross-dockings have been evaluated on the basis of the lowest RMSD values of the ligands (Table S1). The cross-docking experiments revealed that 5ZGE showed the lowest RMSD value (0.30), and it was found to be the optimal structure of the NDM-1 enzyme that could take up new ligands. Overall, the percentage of good and close poses of native ligands was found to be 83% which is an ideal value for the validation of docking protocols. Hence, the structure 5ZGE (PDB ID) of NDM-1 was found to be an optimal structure; hence, it was selected for the screening purpose.

### 2.2. Pharmacokinetics and Screening of Receptor–Ligand Interactions

Based on the lowest RMSD determined through cross-validation (Table S1), the 3D structure of NDM-1 with PDB code 5ZGE (resolution of 1.0 Å) was taken for docking studies. A library of approximately 58,900 compounds was generated for virtual screening with the target protein. For the drug likeness study of the compounds, their physiochemical properties, pharmacokinetic properties, toxicity profile, and synthetic availability were predicted by ADMETlab2.0 [40]. Out of 58,900 compounds, 6925 were found to be suitable for drug designing according to Lipinski’s rule of five (Table S2). The pharmacokinetic properties of the 6925 compounds were further evaluated to ensure that they were within acceptable limits. Among these 6925 compounds, 132 compounds showed acceptable results of the drug likeness test (Table S3). To further examine how these substances interact with the target protein, protein–ligand docking was conducted. Out of the screened compounds, 106 compounds were selected for further investigations primarily based on the least binding energy (meeting the threshold values −7.0 kcal/mol) and the best conformation (Table S3). Other docking analysis parameters were considered, such as zinc–ion interactions, the hydrogen bond count, pi–pi interactions, T-shaped pi–pi interactions, and critical residue interactions. Some compounds were not included in subsequent studies because of the weak interactions (only alkyl interactions). Eventually, a total of four compounds were selected based on protein–ligand interaction (Table 1), and these potential compounds were subjected to further studies. 

These four compounds were found to be non-mutagenic and non-carcinogenic, as determined by the Ames test. Moreover, the oral toxicity class was also determined for the potent compounds. In this regard, the Center for Drug Evaluation and Research (CDER) has designated four categories, with category I designating substances that are poisonous or irritant, and category IV designating substances that are neither toxic nor irritant. Out of these four compounds, monodemethylcurcumin was in toxicity class IV, whereas three compounds (butein, butyl 3-O-Caffeoylquinate, and rosmarinic acid) were in toxicity class III. However, butyl3-O-Caffeoylquinate was found to be damaging to the liver; hence, it was not further investigated. The remaining three compounds (butein, monodemethylcurcumin, and rosmarinic acid) with high gastrointestinal absorption, non-AMES toxicity, no liver toxicity, and no violations of Lipinski’s guidelines were chosen for future study based on the findings of the pharmacokinetic analysis (Table 1).

### 2.3. Predicted Ki Values of Potent Metabolites and Their Comparison with Reported Synthetic Inhibitors

The Ki values of the potent metabolites were predicted through AutoDock4.2. All of the three selected predicted compounds showed a Ki value of less than 100 µM (Table 1). An inhibitor showing a Ki value of less than 100 µM was considered good with reference to its binding affinity toward the receptor [44]. The predicted binding energies of almost all compounds were better than those of the previously reported synthetic inhibitors, including Aspergillomarasmine (−7.36 kcal/mol), Tiopronin (−7.94 kcal/mol), Thiorphan (−7.04 kcal/mol), Dimercaprol (−3.10 kcal/mol), and D-captopril (−6.63 kcal/mol) (Table 1). Among the predicted compounds, butein had the binding energy (−7.8 kcal/mol) and Ki value (2.04 µM), followed by monodemethylcurcumin (−9.3 kcal/mol and 3.33 µM) and rosmarinic acid (−8.9 kcal/mol and 7.35 µM) (Table 1). 

### 2.4. Molecular Interactions and Binding Mode of Potential Metabolites with NDM-1

It is important to investigate whether ligands interact with the key amino acid residues of an enzyme, because this interaction determines whether the ligand can inhibit the enzyme. For this purpose, the 2D structures of the protein–ligand complex were thoroughly investigated. It was found the rosmarinic acid forms hydrogen bonds with the amino acids Asp124, His122, and Glu152 and pi–sulfur interactions with the amino acid Cys208. It also has metal–acceptor interactions with the zinc ions Zn303 and Zn301 (Figure S1). Butein forms hydrogen bonds with the amino acids Asn220, His250, Lys211, and Ser251 and a pi–alkyl interaction with the amino acid Ile35. It also has a metal–acceptor interaction with the zinc ion Zn303 (Figure S2). Monodemethylcurcumin forms hydrogen bonds with the amino acids Gln123, Asp124, Lys211, and Asn220 and pi–alkyl interactions with the amino acids Val73 and Phe70. It also has a pi–donor hydrogen bond with the amino acid His250, a carbon–hydrogen bond with the amino acid His189, and a metal–acceptor interaction with the zinc ion Zn301 (Figure S3).

### 2.5. Molecular Dynamics Simulations

**Figure 1:**
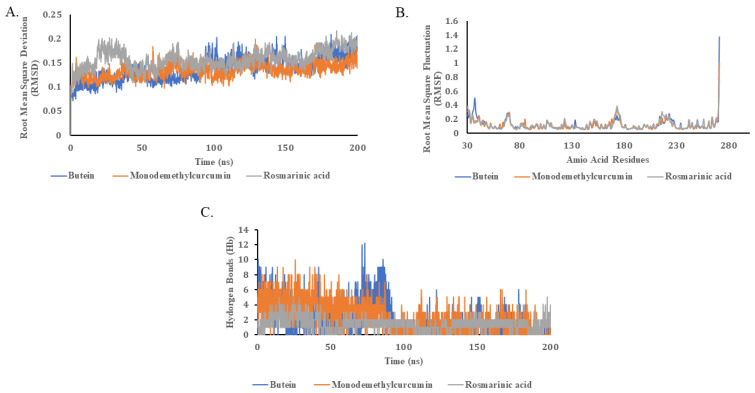
Molecular dynamics trajectory analysis of ligand-protein complex. (**A**) Root mean square deviation (RMSD) value of protein backbone and ligand over a period of 200 ns. (**B**) Root mean square fluctuations of C-alpha of protein due to binding of ligands (**C**) Total number of hydrogen bonds between ligands and protein.

Molecular dynamics (MD) simulations were applied to carefully investigate the binding kinetics of three important ligands (namely, butein, monodemethylcurcumin, and rosmarinic acid) across a period of 200 ns. These simulations were aimed at capturing the extent of dynamic interactions between the ligands and the target protein. Remarkably, all three ligands exhibited minimal deviation, with RMSD values of less than 0.1 Å, as illustrated in Figure 1A. The low RMSD values serve as a strong indicator of the stability and specificity of these ligand–protein complexes. Essentially, a lower RMSD value underscores that the ligands have found a ‘comfortable’ binding pocket within the protein, thus minimizing their positional shifts during the 200 ns simulation.

**Figure 2:**
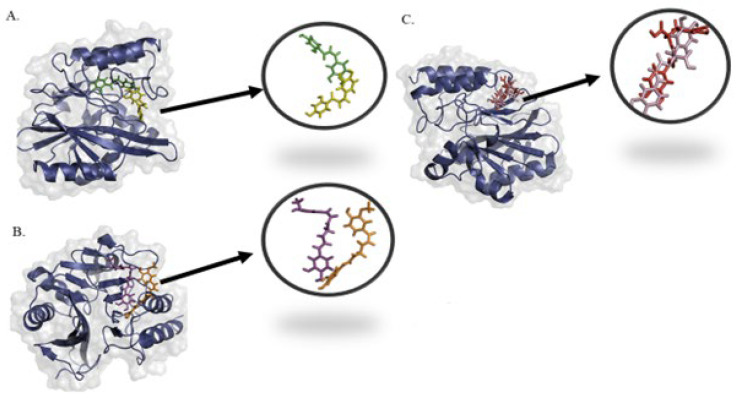
Binding confirmation of selected ligands with the protein at start and end steps of molecular dynamics simulations. (**A**) Butein structural deviation at binding site at 0 ns (in green) and 200 ns (in yellow) (**B**) Monodemethylcurcumin structural deviation at binding site at 0 ns (in magenta) and 200 ns (in orange) (**C**) rosmarinic acid structural deviation at binding site at 0 ns (in red) and 200 ns (in pink).

This notion is also validated when the first and last frame of the molecular dynamics trajectories were visualized (Figure 2). The three ligands showed small structural deviations in their binding pockets. This suggests not only a stable interaction but also implies that these ligands could be strong candidates for drug development, warranting further experimental validation.

In regard to the RMSF (root mean square fluctuation) values, all of the examined protein–ligand complexes displayed strikingly similar behaviors. Noteworthy fluctuations in the RMSF values were observed within specific amino acid residue ranges: 67–72, 171–176, and 214–228, as depicted in Figure 1B. The significance of these RMSF fluctuations cannot be overstated. The active site of NDM-1 is composed of three pivotal loops—L3 and L10, to be precise [43]. Among these, L3 has been previously implicated in substrate specificity and binding [45,46]. The amino acid residues Lys171 and Asn180, located in L10, also play a crucial role in substrate binding [46]. Recent studies have highlighted the importance of residues like Ser217, Gly219, and Asn220 in biological interactions [34]. Elevated RMSF values in these specific regions suggest that these amino acid residues are highly dynamic and likely participate in the critical interactions with the ligands. These dynamic regions might serve as adaptable docking points or as regions that could undergo conformational changes upon ligand binding, thereby affecting the overall stability and function of the protein–ligand complex.

By accounting for the overall number of hydrogen bonds produced between ligands and proteins, the hypothesis is bolstered even further. All of the three ligands formed a similar number of hydrogen bonds throughout 200 ns of the MD simulation (Figure 1C). The consistent formation of hydrogen bonds throughout the simulation signifies a stable interaction, reinforcing the low RMSD values previously discussed. Hydrogen bonds are key elements in maintaining the structural integrity of biological complexes. A consistent number of such bonds implies that the ligands are not only fitting well into the protein’s active site but also maintaining a stable network of interactions. This could be indicative of a strong, long-lasting binding affinity between the ligand and the protein, making these ligands prime candidates for further study and potential drug development. 

**Figure 3:**
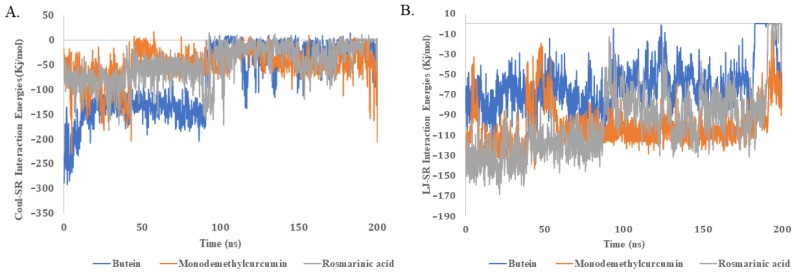
Coul-SR (**A**) and LJ-SR (**B**) binding energy values of ligands with protein. The analysis was performed for a 200-nanosecond duration. The phytochemicals butein, monodemethylcurcumin, and rosmarinic acid were included in the investigation.

We also analyzed the molecular dynamics (MD) trajectory of the protein–ligand complex to calculate the binding energy values. The Coulomb–Schrödinger (Coul-SR) and Lennard–Jones–Schrödinger (LJ-SR) binding energy values of the ligands with the proteins were calculated (Figure 3, Table 2).

All three compounds showed comparable Coul-SR and LJ-SR interactions energies, indicating their comparable binding affinities with the protein. The Coul-SR values offer understandings of the electrostatic interactions between the ligands and the proteins, which are fundamental in stabilizing the complex. A consistent Coul-SR value across the ligands indicates that they all have comparable electrostatic interactions with the protein, suggesting that they are equally efficient in establishing a stable complex. On the other hand, the LJ-SR values provide information about van der Waals forces, which are critical for the ‘fit’ of the ligand within the protein’s binding pocket. Comparable LJ-SR values signify that the ligands are equally adept at snugly fitting into the binding pocket, which is an essential attribute for potent ligand candidates. The similar Coul-SR and LJ-SR interaction energies across the ligands further support their comparable binding affinities with the target protein. This consistent energy profile, coupled with the previously discussed low RMSD and stable hydrogen bonding, makes these ligands compelling candidates for further research and possible drug development.

## 3. Discussion

β-lactam antibiotics are effective against a wide range of bacterial infections, but their effectiveness is challenged by the growing prevalence of β-lactamases, especially NDM-1. This enzyme can break down the key β-lactam ring of a wide range of antibiotics. Carbapenems are among the most powerful antibiotics available, and they are often used as a last resort to treat infections caused by bacteria that are resistant to other antibiotics [23]. Despite the fact that many NDM-1 inhibitors have been developed [47,48,49,50,51], none have been approved for clinical use [7]. Among these, Captopril was discovered to be a potential NDM-1 inhibitor, as the thiol group in this drug has the potential to chelate zinc ions [19,52]. Similarly, Aspergillomarasmine (AMA) inhibits NDM-1 by releasing a second zinc ion from the active site of the enzyme, but its great hydrophilicity also prevents its further development [24]. Brem et al. investigated the efficacy of D-captopril and L-captopril as NDM-1 enzyme inhibitors; however, it is critical to note down the unexpected harmful effects due to cross-reactivity with human metallo-enzymes [25,28]. Furthermore, mutations in allosteric site residue sequences in some MBLs can significantly reduce the need for zinc ions in the enzymes, indicating that focusing solely on metal chelators is not the best approach [53]. Recently, research has highlighted the role of magnolol, a natural compound, as an effective NDM-1 inhibitor. Magnolol is a less toxic compound derived from plants. Researchers have proposed that combining magnolol or other plant-based inhibitors with existing antibiotics could be an effective way to combat antibiotic resistance [19]. Similarly, the efficacy of antibiotics was restored when used in combination with other inhibitors [25,54]. Flavanol compounds (such as quercetin, myricetin, and morin) have also been identified as potent NDM-1 inhibitors [55].

One of the most efficient techniques is to screen inhibitors from known small molecules, i.e., natural compounds with demonstrable biological action, and lead compounds targeting antibiotic resistance enzymes. The idea of combination therapy, i.e., a β-lactam antibiotic and a synthesized inhibitor, are proved to be successful against NDM-1 [3]. The development of an inhibitor for the inhibition of NDM-1 and to restore the efficacy of existing β-lactam antibiotics has become a pressing therapeutic necessity [56].

Several plant species and their metabolites have been used effectively against the antibiotic resistance conferred by serine-based class and resistance through the penicillin-binding proteins. In vivo and in vitro studies against β-lactamase have been observed by using the extracts of edible plants [57]. This led us to explore various edible as well as other plant sources with reported properties against antibiotic resistance through NDM-1. Virtual screening was performed through AutoDock as a docking engine, which helped to identify the best metabolites that could inhibit NDM-1. Synthesized inhibitors against NDM-1, such as Aspergillomarasmine, Tiopronin, Thiorphan, Dimercaprol, and D-captopril, have been taken as a reference to compare the important parameters, such as binding energy, with metabolites predicted in the current study [25,26,42,58]. Upon consideration of the toxicity class and the AMES mutagenesis, our predicted metabolites are found to be non-AMES mutagenic, and they fall in toxicity classes III and IV. Furthermore, AutoDock4 was used to obtain the Ki values of the hit compounds, as a low Ki value implies high potency of the ligand with a range in the micro molar to be qualified as a hit or lead candidate [59]. While comparing the docking scores of reported inhibitors, it has been observed that energy values of our predicted metabolites, i.e., butein, rosmarinic acid, and monodemethylcurcumin, were closely related to the reported ones. In one of the reported in vivo studies, the compound 4,5-Dicaffeoyl quinic acid was proved to be an inhibitor of pigmentation by reducing melanin synthesis [60], and narirutin, which is found in citrus peels, possesses anti-cancer activity along with other metabolites [61]. Three other phytochemicals—coriandrinonediol, oleanderolide, and uzarigenin—were discovered to be highly effective NDM-1 inhibitors in another recent investigation [62]. Following molecular docking, it was determined that their binding affinities to NDM-1 ranged from −8.3 kcal/mol (coriandrinonediol) to −8.1 kcal/mol (uzarigenin) [62]. Similarly, withaferin A, diosgenin, and beta-sitosterol have been proposed as possible inhibitors of NDM-1 based on the binding energies and molecular stability [36]. In another recent investigation, ten phytochemicals were discovered as broad-range MBL inhibitors. One of these phytochemicals, C_30_H_22_O_13_, with the ChEMBL ID CHEMBL3422281, displayed extremely low binding energy, measuring −22.7 kcal/mol [34]. 

On the basis of the aforementioned predictions, the lowest binding energies, the molecular interactions with active site residues, and the molecular dynamics and simulations of the compounds, butein, rosmarinic acid, and monodemethylcurcumin were found to be good candidates for future drugs to inhibit NDM-1 and prevent the β-lactam antibiotics from hydrolysis. 

The important active site residues in NDM-1 have been revealed in various prior structural investigations. For instance, Ile35, Met67, Val73, Trp93, Cys208, Asn220, and His250 were identified by Wang and colleagues as being important NDM-1 residues [63]. Similarly, Met67, Phe70, Lys211, Lys216, Ser217, and Asn220 were identified by Kar and colleagues as crucial residues for the interaction of an enzyme and a substrate. [62]. In a separate investigation, NDM-1 was docked with 16 commercially available antibiotics or inhibitors. The binding energies of the enzyme–substrate complex ranged from −5.20 kcal/mol (imipenem) to −8.53 kcal/mol (meropenem) [36]. During the investigation, it was observed that meropenem formed hydrogen bonds with amino acid residues Gln123, Asp124, Lys211, Asn220, and His250 of NDM-1. Additionally, the importance of amino acid residues Ile35, His122, Val173, His189, Cys208, and Gly219 was also established, as these residues were engaged in hydrophobic interactions [36]. The compounds that were discovered in the current study were found to interact with most of the abovementioned amino acid residues. 

Rosmarinic acid has previously been linked to a number of pharmacological effects, including being anti-inflammatory [64] and preventing acute myocardial infarction via controlling Ca^2+^ homeostasis and plasma antioxidant enzymes [65]. Its application also led to the regeneration of the liver [66] and the protection of the liver against cholestasis [67]. Rosmarinic acid was found to be safe in tests involving cell lines and zebrafish embryos for cytotoxicity and genotoxicity. In a recent experiment, it was revealed that rosmarinic acid strongly inhibited VIM-2 metallo-β-lactamases, while NDM-1 was weakly inhibited [49]. It is interesting to note that NDM-1 was severely suppressed by salvianolic acid A, a rosmarinic acid derivative [49]. It has already been documented that butein has anti-cancer properties [68,69]. Studies have demonstrated that it has a neuroprotective effect against damage brought on by H_2_O_2_ [45]. Interestingly, because it can mask the *h*ACE2 receptor, butein has been predicted as a potential hit phytochemical to block SARS-CoV-2 entry into human cells [70].

Based on current study, it is suggested that the compounds butein, rosmarinic acid, and monodemethylcurcumin can be further optimized as leads in the drug discovery process to develop future drug candidates against NDM-1.

## 4. Materials and Methods

### 4.1. Ligand Selection and Preparation

A detailed literature survey has been conducted for the compilation of plant metabolites that have been reported for the inhibition of different classes of β-lactamases. Compounds from the various phytochemical classes, including flavonoids, tannins, glycosides, saponins, coumarins, terpenoids, alkaloids, and polyphenols, were obtained from FooDB (https://www.foodb.ca (accessed on 18 May 2022)), Phenol-Explorer (http://phenol-explorer.eu (accessed on 10 March 2021)) [29], and IMPPAT, the Indian medicinal plant database (https://cb.imsc.res.in/imppat (accessed on 6 April 2021)) [30].

More than 58,900 compounds were retrieved from the aforementioned databases and collected from the reported literature survey based on their antibacterial properties. The names or SMILES of the ligands were searched in various databases, including the PubChem–NCBI database [71,72] accessible at (https://pubchem.ncbi.nlm.nih.gov/ (accessed on 27 June 2022)), and the ZINC database (https://zinc.docking.org/ (accessed on 27 June 2022)) [73], in order to obtain chemical structures of the ligands in a structured data format (SDF). Compounds whose structures were not listed in these databases had their diagrams created using the stand-alone program ChemDraw. In order to prepare the ligands for docking, SDF formats of the ligands were further translated to Protein Data Bank (PDB) forms. Using Linux instructions for AutoDock Vina, each ligand was treated to 3D protonation and energy minimization. All of the ligand was eventually saved in pdbqt format.

### 4.2. Drug-Likeness Prediction Using ADMETlab2.0

All compounds acquired from various databases must pass a drug likeness test in order to conduct docking experiments. The prediction of drug likeness properties for all compounds was carried out using ADMETlab2.0 [40]. ADMETlab2.0 is a quick, accurate, and easy to use program for the prediction of Absorption, Distribution, Metabolism, Excretion, and Toxicophoric (ADMET) properties. In addition to ADMET properties, this program evaluates many pharmaceutical properties of compounds, like mutagenicity, carcinogenicity, and physiochemical properties of the compounds. This program evaluates the compounds based on different rules of pharmaceutical companies, like Lipinski’s rule [74], which states that the molecular weight of compounds for drug should be <500 Da. The H bond donor should be less than <5, the H bond acceptor should be <10, the number of rotatable bonds should be less than <5, and the log *p* value should be <5. For a compound to pass the carcinogenic and mutagenic property of drug likeness test, the probability value for this compound should be <0.5. Compounds with the greatest number of drug-like properties within the standard value range were chosen for further docking studies. The pkCSM server was used to forecast the liver toxicity of phytochemicals [75].

### 4.3. Protein Selection and Preparation

The selection of suitable molecular targets is a crucial step in docking studies; hence, the 3D structures of NDM-1 were retrieved from the protein data bank (PDB) in PDB format (PBD IDs: 4RL2, 6NY7, 6O3R, 4EYB, 6TWT, 5ZGE). The native ligand was observed from the target protein. The MGL Tool version 1.5.7 (Molecular Graphics Laboratory Tool) of AutoDock Vina was used for the preparation of the protein for docking. The MGL Tool was used for the removal of crystallographic water molecules, the addition of the polar H, along with the distribution of Kollman charges. Finally, the modified structure of the protein was saved in the pdbqt file format. Knowing the binding site before the docking process improves the docking efficiency dramatically; hence, BIOVIA Discovery Studio Visualizer was used to specify the potential ligand binding site in the target protein. 

### 4.4. Validation of Target Protein–Ligand Complexes

For the validation of the docking protocols, native ligands from different crystallographic structures of NDM-1 were taken (Table S1). Native ligands, i.e., 3S3 from the structure 4RL2: PDB, L8J from 6NY7: PDB, XJE from 6O3R: PDB, 0WO from 4EYB: PDB, EPE from 6TWT: PDB, and Z27 from 5ZGE: PDB, were taken.

Cross-dockings were performed, where each native ligand was docked to other available structures of NDM-1 in the protein data bank and the quality of best fit was predicted on the basis of ligand RMSD values (Table S1). This approach is said to be valid if the obtained RMSD value is ≤ 2.0 Å so that the test compounds can be docked with a target protein within same binding site.

### 4.5. Receptor–Ligand Docking and Evaluation of Docking Results 

The following stage involved testing a library of possible compounds against the NDM-1 protein’s active site residues. The input files necessary for AutoDock Vina were created and used for this purpose. The notepad file with the AutoDock Vina Perl script was obtained. Additionally, the MGL tool was used to determine the size of the grid box. In AutoDock, the grid box size was maintained at 76, 94, and 82 for the X, Y, and Z dimensions. The default settings for the energy range and exhaustiveness were maintained at 4 and 10, respectively. The AutoDock Vina developers’ shell script was used to implement Vina files. With Kcal/mol as the unit, the binding affinity of ligands was seen as a negative score. The AutoDock Vina script produced ten poses with distinct binding energies for each ligand. Using PyMOL, the ligand’s position with the highest binding affinity was retrieved from the docked complex and stored in a complex.pdb format. With an emphasis on hydrogen bonds, interactions with zinc ions, pi–pi stacking, pi–sulfur contacts, pi–alkyl interactions, and other favorable interactions, the best docking pose was then visualized using BIOVIA Discovery Studio Visualizer. The residues responsible for substrate specificity identified from the active site were Leu65, Met67, Val73, Gln123,Asp124, Lys211, Asn220, His250, Zn301, and Zn303. On the basis of the best interaction, the final compounds were selected for the prediction of the inhibition constant. The reported synthesized inhibitors, i.e., Aspergillomarasmine, Tiopronin, Thiorphan, Dimercaprol, and D-captopril, against the NDM-1 were taken as a reference to compare the energy values of these reported compounds with the potential metabolites in our study.

### 4.6. Prediction of Inhibition Constant of Selected Compounds Using AutoDock 4.2

The final hit compounds were subjected to AutoDock4 [76] for the prediction of Ki values. In AutoDock4, both the receptor and ligands files were saved in a PDBQT format. The grid box was set and the AutoDock files, i.e., autodock4.exe and autogrid4.exe, were also copied from the program files in the C drive to the working directory. Immediately after the execution, the output files for the evaluation were automatically generated in the gpf (grid parameter file) and dpf (docking parameter file) files.

### 4.7. Molecular Dynamics (MD) Simulations

The dynamic bindings of the top three ligands with the protein were assessed using MD simulations, which were performed using GROMACS 5.0.5 [77]. The CHARMM-36 all atoms force field was used to prepare the topology file of the protein [78]. For the preparation of the topology files of the ligands, CGenff internet service was applied [79]. For the solvation of the protein–ligand complex, a TIP3P water system was used. The solution was neutralized by adding the appropriate amount of Na and Cl ions. The energy minimization was carried out to stabilize the system using the gradient descent optimization algorithm [80]. Before 200 ns of the production phase, 100 ps of NVT and NPT equilibration was carried out. After the production phase, the trajectory was analyzed for the root mean square deviation (RMSD), root mean square fluctuations (RMSF), the total number of hydrogen bonds, and the binding energies of ligands and the protein during simulations.

## 5. Conclusions

Due to the rapid evolution and dissemination of antibiotic resistance genes, there is an urgent need for time to develop new antibiotics, especially against β-lactamases. A significant pool of prospective antibiotic possibilities is offered by phytochemicals. Three compounds—butein, rosmarinic acid, and monodemethylcurcumin—were identified in the current study as strong inhibitors of NDM-1 β-lactamase. The outcomes were further supported by a molecular dynamics simulations study of the structural stability of protein–ligand complexes. In order to create new antibiotics to combat bacterial strains resistant to β-lactams, further research into the recently identified chemicals is necessary.

## Figures and Tables

**Table 1:** Comparison of drug-like properties of predicted metabolites and previously synthesized inhibitors of NDM-1.

Compound Name	Binding Energy (kcal/mol)	Ki (µM)	LogS ^1^	LogP ^2^	Ames ^3^	Carcinogenicity	Toxicophores	Synthetic Accessibility	Lipinski	Reference
Butein	−9.1	2.04	−2.99	2.63	0.826	0.509	3	2.22	Accepted	This Study
Monodemethylcurcumin	−9.3	3.33	−3.33	2.57	0.289	0.599	3	2.49	Accepted	
Rosmarinic acid	−8.9	7.35	−2.43	1.77	0.035	0.275	3	2.90	Accepted	
Aspergillomarasmine	−7.36	508.94	−2.15	−5.94	0.013	0.067	3	3.42	Rejected	[25]
Tiopronin	−7.94	586.05	−0.51	−0.47	0.015	0.03	3	2.92	Accepted	[41]
Thiorphan	−7.04	1.25	−1.73	1.23	0.603	0.117	3	2.44	Accepted	[41]
Dimercaprol	−3.1	510.9	−0.54	0.59	0.814	0.931	2	4.39	Accepted	[42]
D-captopril	−6.63	600.3	−0.61	0.27	0.01	0.029	3	3.03	Accepted	[43]

^1^ Aqueous solubility value. ^2^ Octanol/water distribution coefficient. ^3^ Values ranging from 0 to 0.3 are considered acceptable. In case of carcinogenicity, values ranging from 0 to 0.5 are considered acceptable. The class one and two of toxicophores are considered highly toxic. The lower the value of synthetic accessibility, the easier it is to synthesize a compound.

**Table 2:** Coul-SR and LJ-SR values of the investigated phytochemicals.

Names of Compounds	Average Energy Interactions kJ.mol^−1^ (Coul-SR)	Average Energy Interactions kJ.mol^−1^ (LJ-SR)
Butein	−77.533	−61.658
Monodemethylcurcumin	−46.918	−98.748
Rosmarinic acid	−50.221	−101.427

## Data Availability

Data is contained within the article and the Supplementary Materials.

## Supplementary Materials

The following supporting information can be downloaded at: https://www.mdpi.com/article/10.3390/ph16101404/s1. Table S1. Structures of NDM-1, their native ligands, and predicted RMSD values. Table S2. Pharmacokinetics and physiochemical analysis of phytochemicals. Table S3. List of phytochemicals showing acceptable values of pharmacokinetics and Lipinski’s rule of five. Figure S1. The 2D and 3D representations of binding of rosmarinic acid with the protein. Figure S2. The 2D and 3D representations of binding of butein with the protein. Figure S3. The 2D and 3D representations of binding of monodemethylcurcumin with the protein. Reference [81] is cited in the supplementary materials.
