# Supplementary material for: Prediction of Phytochemicals for Their Potential to Inhibit New Delhi Metallo β-Lactamase (NDM-1)
Source: Pharmaceuticals (Basel). 2023 Oct 3;16(10):1404. doi: 10.3390/ph16101404 (PMC10610165; doi:10.3390/ph16101404)
Supplement: Supplementary file 1 [file pharmaceuticals-16-01404-s001.zip › Figures S1-S3.pdf]

Rosamarinic acid

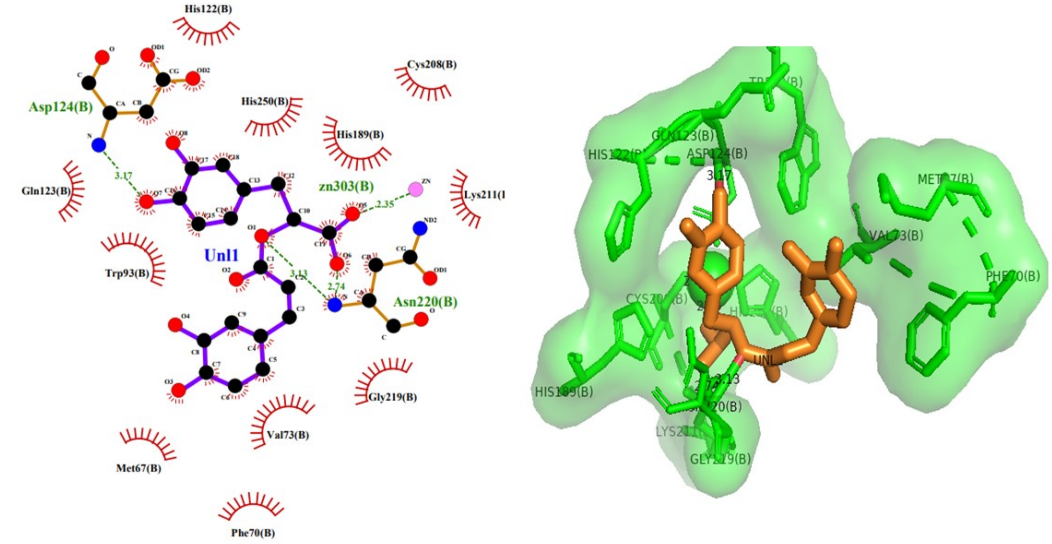

Figure S1. The 2D and 3D representations of binding of rosmarinic acid with the protein.

Butein

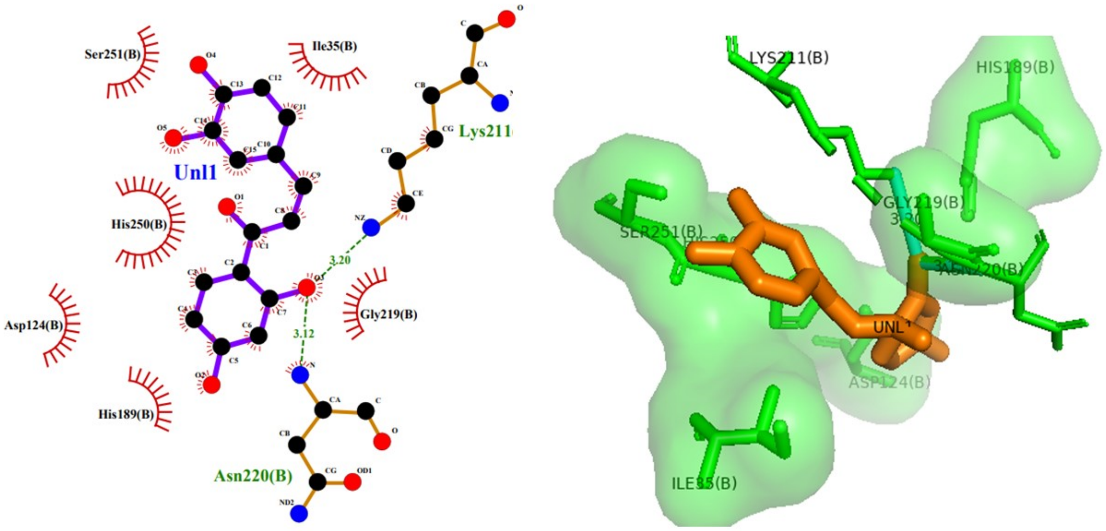

Figure S2. The 2D and 3D representations of binding of butein with the protein.

Monodemethylcurcumin

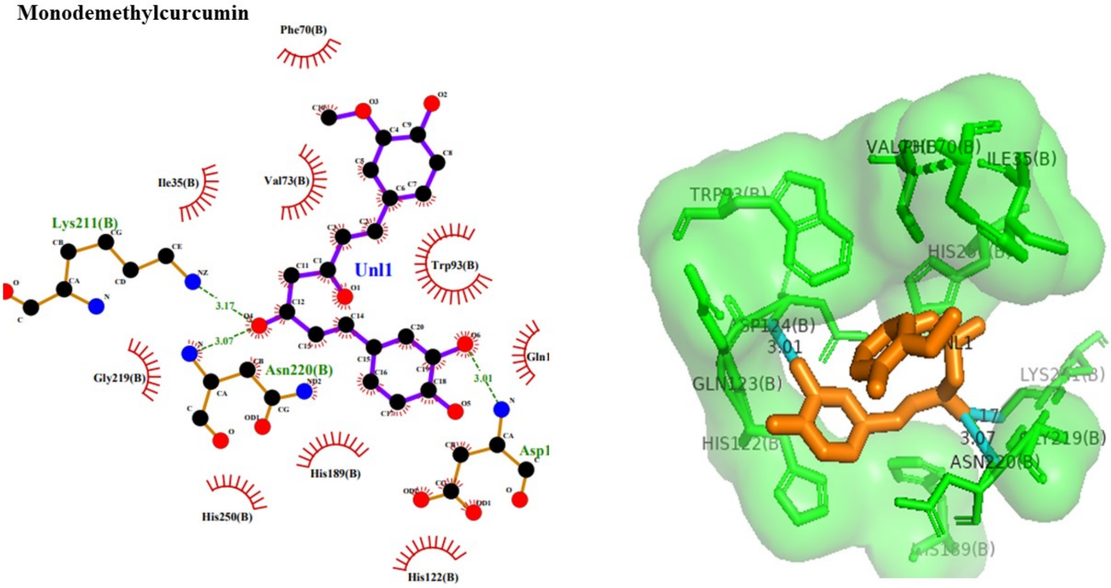

Figure S3. The 2D and 3D representations of binding of monodemethylcurcumin with the protein.
