# Supplementary material for: Prediction of Phytochemicals for Their Potential to Inhibit New Delhi Metallo β-Lactamase (NDM-1)
Source: Pharmaceuticals (Basel). 2023 Oct 3;16(10):1404. doi: 10.3390/ph16101404 (PMC10610165; doi:10.3390/ph16101404)
Supplement: Supplementary file 1 [file pharmaceuticals-16-01404-s001.zip › Table S1.pdf]

Table S1. Structures of NDM-1, their native ligands and predicted RMSD values

Cross validation of native ligands from crystallographic structures of NDM-1 based on RMSD values							
		**4RL2**	**6NY7**	**6O3R**	**4EYB**	**6TWT**	**5ZGE**
**Native ligands**	**3S3**	3.19	3.2	2.50	1.20	2.68	3.29
	**L8J**	1.50	2.87	1.76	1.13	1.50	1.61
	**XJE**	2.10	0.68	1.55	1.10	1.20	0.70
	**0WO**	3.20	3.15	4.46	2.55	1.02	1.52
	**EPE**	0.80	0.88	0.92	0.81	0.78	0.60
	**Z27**	1.11	1.21	1.47	1.34	1.97	0.30

Following criteria has been adopted as reported in study (Bajda et al., 2013).

RMSD ≤ 1.0 Å, good pose. (Green)

RMSD ≤ 2.0 Å, close pose. (Blue)

RMSD ≤ 3.0 Å, pose with errors. (Red)

RMSD >3 .0 Å, bad pose. (Pink)

Bajda, M., Więckowska, A., Hebda, M., Guzior, N., Sotriffer, C. A., & Malawska, B. (2013). Structure-based search for new inhibitors of cholinesterases. International Journal of Molecular Sciences, 14(3), 5608-5632.
